# The Investigation of the Effect and Mechanism of* Sophora moorcroftiana* Alkaloids in Combination with Albendazole on Echinococcosis in an Experimental Rats Model

**DOI:** 10.1155/2018/3523126

**Published:** 2018-03-28

**Authors:** Fabin Zhang, Chunhui Hu, Shilei Cheng, Shulin Wang, Bin Li, Deping Cao, Haining Fan, Ruchong Pan, Mei Yang, Yanhui Xu

**Affiliations:** ^1^Medical College of Qinghai University, Xining 810001, China; ^2^Qinghai Institute for Endemic Disease Prevention and Control, Xining 811602, China; ^3^Qinghai University Affiliated Hospital, Xining 810016, China

## Abstract

Echinococcosis is a worldwide anthropozoonosis which is highly endemic over large animal husbandry areas in northwestern China. The current clinical therapeutic medicine against echinococcosis is albendazole, although it caused serious side effects in patients. The component in traditional Chinese herb medicine,* Sophora moorcroftiana* alkaloids (SA), is thought to be a potential drug to treat echinococcosis. In order to explore the effect and mechanism of SA treatment against echinococcosis, we established animal echinococcosis model and treated rats with albendazole alone, alkaloids alone, and combined therapy. The combined treatment showed effective inhibition against parasite infection due to induction of host response and alleviated liver injury; meanwhile albendazole caused serious liver problem. The proteomics study revealed that the combined therapy might induce complement activation through C3, C4, C5, SERPINA1, and SERPINC1 proteins and cell adhesion by ANXA2, EZR, YWHAB, HSP90AN1, and PRKAR2A proteins, while albendazole treatment could induce liver injury through CRYAB, YWHAZ, SLC25A24, and HSPA1B proteins that were involved in cell death. In all, we consider that the combinational treatment displayed better therapeutic effects against liver echinococcosis as well as alleviated liver injury, which could be considered as an effective strategy to treat echinococcosis clinically.

## 1. Introduction

Echinococcosis is a worldwide anthropozoonosis which is caused by* Echinococcus granulosus* [1]. In China, it is highly endemic over large animal husbandry areas in northwestern provinces. As estimated, approximately one percent of the farmer population in these areas was infected by* Echinococcus granulosus*. In humans, ingested eggs can be mainly distributed to liver and lung, leading to cystic echinococcosis (CE) and alveolar echinococcosis (AE). CE infection is the leading consequence, which is responsible for over 98 percent of all echinococcosis cases [2]. The current clinical treatment strategies against echinococcosis are surgery and chemotherapy; other approaches including gamma-ray treatment are still limited to bench level [2, 3]. However, surgery is prone to cause parasite lesion residual or unfortunate parasite dissemination by inappropriate operation, leading to disease relapse. Meanwhile, the use of chemotherapy also does not achieve desirable effect. Albendazole is the most common clinical drug to treat echinococcosis [4]. But it showed poor solubility in gastrointestinal (GI) tract, causing low drug concentration in liver. Albendazole also causes serious adverse side effects in patients such as encephalitis syndrome, influenza-like syndrome, allergic purpura, and drug rash. Furthermore, it has been reported that* Echinococcus granulosus* protoscolices have developed resistance to albendazole [4–6]. Thus, it is urgent to develop new therapeutic strategies against echinococcosis.

*Sophora moorcroftiana*, also known as Tibet* S. viciifolia*, thorn firewood, is an endemic leguminous shrub widespread in valleys of Tibet plateau in China. The decoction of its seeds has been commonly used in folk medicine to treat parasitosis by local people for years. The main composition of alkaloids in the seed decoction includes oxymatrine, sophora, sophorine, and matrine, which was also used as an emetic, detoxicant, and antiphlogistic and in verminosis in traditional Chinese medicine [7, 8]. Clinically, its seeds decoction is combined with albendazole to treat echinococcosis [9]. It was reported that the alkaloids from* Sophora moorcroftiana* is the potential active ingredient in this folk medicine [7, 10]. In the present study, we not only investigated the therapeutic effect of the combinational treatment of* Sophora moorcroftiana* alkaloids and albendazole against echinococcosis in an experimental rats model, but also explored the underlying molecular mechanism of this strategy by proteomics. First, we evaluated the effect of combination therapy by measuring several blood biochemical indicators and histological observation; then, we employed quantitative proteomic assays using isobaric tags for relative and absolute quantitation (iTRAQ), combined with high performance liquid chromatography-tandem mass spectrometry (HPLC-MS/MS), to detect proteome alteration in different treatment. Additional bioinformatics analyses were used to analyze the differential proteins (DPs) to investigate the key pathways underlying the mechanism of combinational treatment. The results showed that the combination therapy was effective in treating echinococcosis in animal model. More importantly, it was found that the combination therapy leads to complement activation and elevated cell adhesion, while the treatment with albendazole alone induced cell death which might cause hepatic injury.

## 2. Materials and Methods

### 2.1. Materials

*Sophora moorcroftiana* used in this study was purchased from Linzhi, Tibet. Alkaloids (purity > 90%) were extracted from* S. moorcroftiana* seeds in our laboratory and prepared for use as described previously [9]. Albendazole was purchased from Zhejiang Wanma Pharma Ltd. Co., Hangzhou, China. The RPMI medium, IL-2, IL-6, IL-10, IgE, and TNF-*α*  ELISA detection kits were purchased from Invitrogen, USA. The aspartate aminotransferase (AST) activity assay kit and the alanine transaminase (ALT) activity assay kit were obtained from Sigma-Aldrich, USA.

### 2.2. Protoscolex Collection

*Echinococcus granulosus* protoscolices were kindly provided by Qinghai Institute for Endemic Disease Prevention and Control, China. The protoscolices were aseptically removed from liver hydatid cysts obtained from cattle and washed several times with saline containing 1500 U/mL penicillin and 1000 U/mL streptomycin [11]. The survival rate of the protoscolices exceeded 95% after these procedures.

### 2.3. Animal Study

The experimental animal protocols were approved by the Experimental Animal Care and Ethics Committees of Qinghai University. 54 female Sprague-Dawley rats were purchased from Research Laboratory Center of Gansu University of Traditional Chinese Medicine (Gansu, China). All rats were 10 weeks old with a body weight between 180 g and 200 g (certification number: SCXK (gan) 2011-0001). All rats were randomly divided into two groups, 64 rats in experiment group and 10 rats in normal group. The rats in experiment group were inoculated intraperitoneally with 4,500 viable protoscolices in 0.3 mL RPMI medium, while the rats in normal group were injected intraperitoneally with 0.3 mL normal saline. The rats were housed under standard conditions (temperature: 18–22°C, humidity: 50–60%) with free access to food and water. After 30 days (12), four rats from experiment group were randomly sacrificed for histological observation, in order to ensure successful establishment of echinococcosis animal model.

The 40 infected rats were divided into four groups (10 rats per group). Rats were administered with* Sophora moorcroftiana* alkaloids (SA) alone (SAT group, 8 mg/kg per day, once a day), albendazole (A) alone (AT group, 20 mg/kg per day, once a day), and combined treatment ( SAT + AT group, 8 mg/kg per day SA + 10 mg/kg A per day, once a day) by gavage, respectively. The rats in model group (M group) were given equivalent volume of normal saline. The normal group (N group) of 10 uninfected rats was also treated with normal saline.

All rats were anesthetized and sacrificed under the experimental protocols described above and all efforts were made to minimize animal suffering.

### 2.4. Blood Indicators Examination

Thirty days after treatment, rats were sacrificed and blood was collected. Serum was obtained by centrifugation. The level of IL-2, IL-6, IL-10, IgE, and TNF-*α* was measured by a microplate reader (BioRad, xMark-10483) using ELISA detection kits (Invitrogen, USA). The AST and ALT level in serum were also detected by Sigma-Aldrich kits (USA).

### 2.5. Pathologic Histology Analysis

For pathological analysis, rats were sacrificed and the hydatid cysts were harvested from peritoneal cavity and liver. The thymus and spleen were also collected. The weight of the hydatid cysts, thymus, and spleen was measured, respectively. Thymus index, spleen index, and inhibition rate of cysts were calculated as follows: thymus index = (thymus weight/body weight) × 10; spleen index = (spleen weight/body weight) × 10; inhibition rate of cysts = (the weight of cysts in model group − the weight of cysts in experiment group)/the weight of cysts in model group × 100%.

To observe histological changes after treatment, liver and spleen were collected, sectioned, and stained with hematoxylin-eosin (H&E) staining. Observation was performed under microscope.

### 2.6. Proteomic Analysis

In order to study the molecular mechanism of SA plus albendazole combination therapy treating liver echinococcosis, we performed proteomic analysis on animal samples from five experiment groups (SAT-L group, AT group, SA + AT group, model group, and normal group). One gram of rats liver from each group (0.1 g per rat) was collected for protein extraction. Then, the protein (100 ug) was digested with trypsin for 12 h at 37 °C (protein/enzyme = 100/3.3). After iTRAQ (AB Science) labeling, equal amounts of labeled peptides from each group were mixed and resolved into 15 fractions by high performance liquid chromatography (HPLC), followed by Q Exactive mass spectrometry (Thermo Fisher Scientific). The resulting MS/MS data were qualitatively and quantitatively analyzed by Mascot 2.3.01 with the following parameters: protein identification using nonredundant International Protein Index rat protein database (version 3.72), full trypsin digest with maximum 1 missed cleavage, peptide tol., and MS/MS tol. were 0.05 Da. Scaffold software was used to identify the differential proteins (DPs). Proteins with *P* < 0.05 and fold change higher than 1.2 or lower than 0.833 were DPs.

### 2.7. Statistical Analysis and Data Preprocessing

The data are presented as mean ± standard deviation (SD). Statistical comparisons among experimental groups were made by Student's *t*-tests using SPSS 22.0 software. Difference was considered significant when *P* > 0.05. The GO and KEGG pathway enrichment analysis of DPs were performed using the Database for Annotation, Visualization, and Integrated Discovery (DAVID) [12].

## 3. Results

### 3.1. Blood Indicators Examination

As seen in Table 1, the level of IL-2 increased when SA was given to rats. The IL-2 level in SAT + AT group was significantly higher than that in model group, while no obvious difference was observed between each treatment group and normal group. For IL-6, its amount in SAT group showed significant difference compared with model group, but there is no significant difference among other groups. The level of IL-10 was also increased when albendazole was administered to rats. When combined with SA treatment, the combinational treatment induced the highest IL-10 expression, which was significantly higher than that in model group yet not significantly different from that in normal group. The IgE expression in treatment group was obviously lower than that in model group (*P* > 0.05), but showed no difference compared with normal group. In addition, the level of TNF-*α* among all groups displayed no significant difference.

### 3.2. Pathologic Histology Analysis

As shown in Table 2, the thymus index was increased when SA was administered to rats, but there was no significant difference compared with model group. Similarly, the spleen index was also elevated when SA was given. However, the combinational therapy group displayed significant higher spleen index value than model group, while no obvious difference was observed between each treatment group and normal group. Besides, we detected that the cyst weight in treatment group was significantly lower than that in model group. Moreover, the combined treatment group showed significantly lower cysts weight and higher inhibition rate of cysts than SA alone-treated groups.

**Figure 1:**
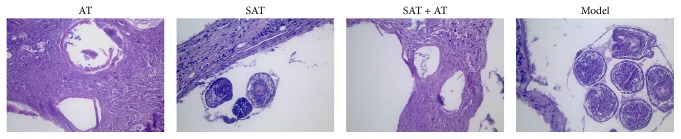
Histological observation of hydatid cysts in each experiment group (magnification, 40x). Different structure of cysts and surrounding tissues could be observed. Sections were stained with H&E.

We also investigated histological changes in hydatid cysts, liver, and spleen tissues after treatment. As shown in Figure 1, the tissues of the hydatid in model group were well developed, and the protoscolex and the intact brood capsule could be found. In SAT group, the structure of the brood capsule was shrinking and collapsing, indicating the therapeutic effect of SAT. In the AT and SAT + AT group, we observed that the nuclear germinal layer cells shrink, dissolved, and even disappeared; no protoscolex structure was detected. Meanwhile, necrosis was observed in the surrounding tissues.

**Figure 2:**
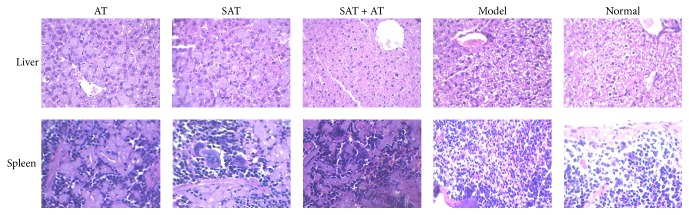
Histological observations. Obvious deposition could be detected in liver and spleen tissue in AT group compared to normal group, while such deposition was attenuated in SAT and SAT + AT group (magnification, 40x). Monocytes and polykaryocytes could be found in spleen tissue in all treatment groups (magnification, 40x). Sections were stained with H&E.

As shown in Figure 2, obvious deposition could be observed in both liver and spleen tissue from AT group; meanwhile such deposition was alleviated in other treatment groups. However, a fair number of monocytes filtrated in spleen tissue and a few polykaryocytes could also be detected in spleen tissue in all treatment groups.

### 3.3. Liver Function Evaluation

With the administration of SA or albendazole, the AST level of rats increased (Table 3). Thereinto, SAT + AT group induced lowest AST expression, but still obviously higher than normal group. It is clear that all treatments caused hepatic injury. As for ALT level, the difference among all groups was not significant. However, the ALT level in combinational therapy group was lowest.

### 3.4. Proteomics Analysis

**Figure 3:**
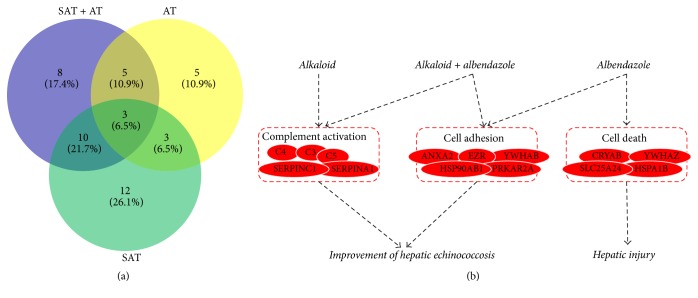
Venny plot of DPs between each group and PPI network of normalized DPs. (a) Venny plot of DPs between each group. (b) Three groups of DPs and potential mechanism of each treatment against echinococcosis.

To explore the underlying molecular mechanism of combination treatment, the liver tissues from five groups were collected for proteomics analysis using an iTRAQ approach. A total of 711 proteins were identified. There were 156 DPs between model and normal group, 126 DPs between SAT group and model group, 123 DPs between AT group and model group, and 138 DPs between SAT + AT group and model group. As shown in Figure 3(a), 67 overlapping DPs were found between the comparison of DPs in control versus model and model versus SAT group, 57 overlapping DPs were found between the comparison of DPs in control versus model and model versus AT group, and 65 overlapping DPs were found between the comparison of DPs in control versus model and model versus SAT + AT group. Further analysis of these overlapping DPs revealed abnormal expression of proteins. There were 38 proteins abnormally expressed in model group and normalized in SAT group (named SAT-normalized DPs), of which 12 DPs were upregulated in model group and downregulated in SAT group and 26 DPs were downregulated in model group and upregulated in SAT group (Table 4). Further investigation of these DPs' biological functions revealed that they were enriched in complement activation process (Table 5). There were 32 proteins abnormally expressed in model group and normalized in AT group (named AT-normalized DPs), of which 12 DPs were upregulated in model group and downregulated in AT group and 20 DPs were downregulated in model group and upregulated in AT group (Table 4). These DPs were found associated with cell adhesion and cell death (Table 5). There were 34 proteins abnormally expressed in model group and normalized in SAT + AT group (named SAT + AT-normalized DPs), of which 18 DPs were upregulated in model group and downregulated in SAT + AT group and 16 DPs were downregulated in model group and upregulated in SAT + AT group (Table 4). These DPs were found involved in complement activation and cell adhesion (Table 5).

We investigated the associated function and potential relationship of the enriched normalized DPs. As shown in Figure 3(b), there were three groups of DPs. C3, C4, C5, SERPINA1, and SERPINC1 and their interaction were found to be involved in complement activation procedure; all of them were downregulated in model group and upregulated in SAT and SAT + AT group. ANXA2, EZR, YWHAB, HSP90AN1, PRKAR2A, and their interactions were also found to be involved in cell adhesion; they were downregulated in model group and upregulated in AT and/or SAT + AT group. Meanwhile, CRYAB, YWHAZ, SLC25A24, and HSPA1B were found associated with cell death; they were downregulated in model group and upregulated in AT group.

## 4. Discussion

Echinococcosis is a widespread zoonosis caused by* Echinococcus granulosus*, which is a very popular disease in large western region of China [1, 7]. Clinically, albendazole was normally used to treat echinococcosis. However, its poor solubility and severe side effects limit its application. In animal husbandry areas of Tibet and some other western provinces of China, people have used decoction of* Sophora moorcroftiana* to treat echinococcosis patients for years. But the mechanism of this Chinese traditional medicine has not been investigated so far. Previous study indicated alkaloids extracted from* Sophora moorcroftiana* were the most effective active ingredients [7, 9]. Thus, it might be a potential way to treat echinococcosis by using* Sophora moorcroftiana* alkaloids in treatment.

In present study, we treated experimental echinococcosis animal model with* Sophora moorcroftiana* alkaloids and also with* Sophora moorcroftiana* alkaloids combined with the clinical medicine albendazole. Compared with albendazole-alone treatment,* Sophora moorcroftiana* alkaloids alone or the combined treatment with albendazole showed obvious therapeutic effects against echinococcosis in infected rats. In* in vivo* study, SAT treated animals showed inhibited cysts development compared with model rats. The cysts weight was significantly reduced by SAT and the inhibition rate was between 30% and 40%. The clinical medicine albendazole had greater effective inhibitory efficacy against echinococcosis infection. The inhibition rate of cysts even achieved 80%. However, the combined treatment was the most potent therapeutic strategy. The rats treated with SAT plus AT showed the lowest cysts weight and the highest inhibition against echinococcosis. In histological observation, we found that echinococcosis infection induced deposition in liver cells, resulting in cells swelling and alveolar wall thickening. When treated with AT alone, this situation was not changed obviously. In contrast, it was attenuated by SAT or SAT + AT treatment. In spleen tissue, it was the same scenario. Large number of lymphocytes filtrated in liver and spleen tissue due to immune response to infection. It caused swelling of infected tissues and increased tissue volume. This result coincided with the comparison of spleen weight in all groups. It revealed that such histological change could be alleviated by SAT + AT therapy.

Cytokines play important roles in host response to infections. Thus we examined IL-2, IL-6, IL-10, IgE, and TNF-*α* level in rats serum. We did not detect significant difference in IL-6 and TNF-*α* expression among all groups. However, the expression of IL-2 in AT group and SAT + AT group was significantly higher than that in model group, while SAT-treated alone group did not show significant difference compared with model group. As an important mediator in inflammatory and immune response of several infectious diseases, IL-2 is able to enhance host immunity and inhibit growth of tumors and parasites [13–15]. Once rats were infected by* Echinococcus granulosus*, the IL-2 receptors mIL-2R in target cells could be overexpressed. The IL-2 level in serum was reduced due to the binding of IL-2 to overexpressed mIL-2R, thus leading to suppression of immune activity of T cells and favoring the parasites survival. Therefore, the expression of IL-2 was increased after treatment, especially in SAT + AT group, which enhanced host immunity against infection, accelerated clearance of polypide, and inhibited parasite growth. IL-10 is a multifunctional cytokine synthesized by Th2 cell subpopulation, which is associated with humoral immunity regulation and host susceptibility to certain disease [16]. Overexpression of IL-10 revealed elevated humoral immunity and suppressed T cells activity, challenging survival of parasites. The IL-10 level in AT group and SAT + AT group was obviously greater than that in model group, but not significantly different compared to normal group. The result indicated that IL-10 played an important part in immune response to echinococcosis infection, although the regulatory mechanism was not clear. It was estimated that IL-10 was associated with Tc cells function, antibody-dependent and complement participative autoimmune response, although the detailed mechanism needs further investigation. Hydatid cysts secrete multiple antigens in host body during its growth and development, which will stimulate different variety of antibodies like Ig G, Ig M, IgA, and IgE. Our result showed that the level of specific antibody IgE in model group was apparently higher, while the IgE level in treated animals was decreased to normal level. It indicated that these treatment strategies exert therapeutic effect against hydatid cyst, which could be considered as an index for evaluating treatment efficacy and prognosis.

We also examined liver function of each group by testing AST and ALT concentration in serum. High level of AST and ALT indicated hepatic injury and liver dysfunction. The model group showed higher AST and ALT level compared to normal group. After treatment, the AST and ALT level in SAT + AT group was decreased while only ALT level in other treatment groups was attenuated. It suggested that echinococcosis infection induced hepatic dysfunction and SAT and AT treatment alone might aggravate hepatic injury (especially AT alone even induced highest AST and ALT concentration) while the combinational therapy ameliorated liver damage [17, 18].

The underlying mechanism was also explored by proteomics analysis. To characterize the molecular mechanism of SAT or AT or SAT + AT treatment, a proteomics analysis was performed to investigate multitargets characteristics. All abnormal expression of DPs was normalized and analyzed, respectively. As shown in Figure 3(b), functional enrichment analysis indicated that these DPs took important part in complement activation, cell adhesion, and cell death. Thus, we explored how these DPs linked to physiological alterations in serum indicators and histological changes in animal tissues. At first, C3, C4, C5, SERPINA1, and SERPINC1 were downregulated in model group and upregulated in SAT and SAT + AT group, which were found to be involved in complement activation procedure. Therein, the three proteins C3, C4, and C5 are key components in complement system, whose activation enhances the ability of antibodies and phagocytic cells to clear antigens, promotes inflammation, and attacks the pathogen's plasma membrane [19]. Alkaloids treatment (SAT group and SAT + AT group) significantly stimulated expression of these complement activation-associated proteins and enhanced host immune response against parasite infection by increasing IL-2 and IL-10 level in serum. Second, albendazole treatment (AT group and SAT + AT group) could upregulate expression of ANXA2, EZR, YWHAB, HSP90AB1, and PRKAR2A, all of which are involved in cell adhesion [20, 21]. The production of cell-adhesion molecules, as well as inflammatory cytokines, was activated by host's macrophages [22]. It is estimated that albendazole treatment may be able to induce activation of macrophage, although the molecular mechanism needs further investigation. These results indicated that the combined treatment with SAT and AT could activate complement system and induce macrophage activation to produce cell-adhesive molecules, leading to improvement of hepatic echinococcosis. On the other side, albendazole treatment alone (AT group) also induced expression of CRYAB, YWHAZ, SLC25A24, and HSPA1B, which were found to be involved in cell death; meanwhile the expression of these proteins was not upregulated in combinational therapy group [23]. It might be able to explain why the liver injury in AT group was obviously more serious than that in SAT + AT group. Albendazole treatment upregulated the expression of these proteins associated with cell death, leading to hepatic injury and liver dysfunction (significantly high level of AST and ALT in AT group), while SAT was able to attenuate tissue damage and loss of liver function [18]. In this scenario, the combinational treatment displayed better therapeutic effects against liver echinococcosis as well as alleviated liver injury, which could be considered as an effective strategy to treat echinococcosis clinically.

## Figures and Tables

**Table 1:** The level of immunological factors in rats serum from different groups.

Group	*N*	IL-2 (pg·ml^−1^)	IL-6 (pg·ml^−1^)	IL-10 (pg·ml^−1^)	IgE (pg·ml^−1^)	TNF-*α* (pg·ml^−1^)
SAT group (8 mg/kg·d)	10	445.25 ± 62.39^#2■1^	750.93 ± 35.08^▼1^	581.27 ± 8.97^#2■2^	182.23 ± 33.07^▼2■2^	539.83 ± 48.66
AT group (20 mg/kg·d)	10	513.42 ± 54.86^▼1^	765.80 ± 48.11^▼2^	606.40 ± 13.12^▼2^	195.71 ± 22.52	567.21 ± 12.04
SAT + AT group (8 mg/kg·d SA + 20 mg/kg·d A)	10	488.45 ± 28.10^▼1^	763.58 ± 33.80^▼2^	606.00 ± 15.49^▼1^	198.19 ± 36.97^▼2^	560.34 ± 17.15
Model group	10	406.96 ± 40.55^*∗*1^	815.12 ± 44.88	581.78 ± 29.19^*∗*2^	232.70 ± 47.15^*∗*2^	557.78 ± 17.11
Normal group	10	489.51 ± 26.35	781.51 ± 33.92	609.28 ± 21.24	183.81 ± 44.32	546.68 ± 25.49

*F* value		8.9538	3.8924	5.5637	2.8967	1.6086
*P* value		0.0000	0.0085	0.0010	0.0324	0.1886

Data were expressed as mean ± SD; ^*∗*^compared with normal group; ^▼^compared with model group; ^■^compared with AT group; ^#^compared with SAT + AT group. ^1^*P* < 0.01; ^2^*P* < 0.05.

**Table 2:** Comparison of pathological indicators in all groups.

Group	*N*	Thymus index (mg·g^−1^)	Spleen index (mg·g^−1^)	Cysts weight (g)	Inhibition rate of cysts (%)
SAT group (8 mg/kg·d)	10	2.03 ± 0.98	4.41 ± 1.97	1.99 ± 1.55^▼2^	34.93 ± 8.94^■2^
AT group (20 mg/kg·d)	10	2.63 ± 0.78	4.45 ± 1.45^#2^	1.40 ± 1.20^▼1^	66.78 ± 15.42
SAT + AT group (8 mg/kg·d SA + 10 mg/kg·d A)	10	2.55 ± 1.26	6.68 ± 2.53^▼2^	0.73 ± 0.60^▼1^	80.39 ± 39.87^■1^
Model group	10	2.95 ± 0.95	4.52 ± 2.04	4.08 ± 3.06	-
Normal group	10	3.49 ± 1.95	3.44 ± 1.46	-	-

*F* value		1.8419	3.8061	6.1815	8.5625
*P* value		0.1374	0.0095	0.0017	0.0013

Data were expressed as mean ± SD; ^▼^compared with model group; ^■^compared with AT group; ^#^compared with SAT + AT group. ^1^*P* < 0.01; ^2^*P* < 0.05.

**Table 3:** Comparison of liver function in all groups.

Group	*N*	AST (ng·ml^−1^)	ALT (ng·ml^−1^)
SAT group (8 mg/kg·d)	10	330.68 ± 47.86^*∗∗*^	545.81 ± 54.51
AT group (20 mg/kg·d)	10	318.57 ± 46.72^*∗∗*^	582.82 ± 73.31
SA + AT group (8 mg/kg·d SA + 10 mg/kg·d AL)	10	272.20 ± 59.54^*∗*^	532.12 ± 84.33
Model group	10	292.90 ± 59.58^*∗∗*^	571.34 ± 54.64
Normal group	10	212.14 ± 45.78	515.43 ± 55.46

*F* value		8.0028	1.7802
*P* value		0.0001	0.1495

Data were expressed as mean ± SD; ^*∗*^*P* < 0.05, compared with normal group; ^*∗∗*^*P* < 0.01, compared with normal group.

**Table 4:** Normalized differential proteins (DPs) in treatment groups.

	Accession number	Symbol	Molecular weight (kDa)	Ratio (model/control)	Ratio (treatment/model)
SAT + AT group	IPI00231139	Tkt	71	0.406126198	1.231144413
	IPI00326305	Atp1a1	113	0.435275282	1.515716567
	IPI00230837	Ywhab	28	0.466516496	1.866065983
	IPI00210542	Gsta4	26	0.5	1.414213562
	IPI00210234	Pls3	71	0.574349177	1.624504793
	IPI00213036	C4b	192	0.707106781	1.741101127
	IPI00470288	Ckb	43	0.707106781	1.231144413
	IPI00471584	Hsp90ab1	83	0.707106781	1.515716567
	IPI00213463	Actn4	105	0.757858283	1.231144413
	IPI00325146	Anxa2	39	0.757858283	1.319507911
	IPI00231136	Nid1	137	0.757858283	1.741101127
	IPI00191761	Rab5c	23	0.757858283	1.231144413
	IPI00327469	Ahsg	38	1.231144413	0.757858283
	IPI00230787	Car2	29	1.231144413	0.812252396
	IPI00200352	Crip2	23	1.231144413	0.659753955
	IPI00421517	Des	53	1.319507911	0.812252396
	IPI00231925	Gnai2	41	1.319507911	0.535886731
	IPI00189809	Myh6	224	1.319507911	0.707106781
	IPI00208061	Atp1b3	32	1.414213562	0.615572207
	IPI00190240	Rps27a	18	1.414213562	0.757858283
	IPI00200466	Slc25a5	33	1.414213562	0.812252396
	IPI00191444	Capzb	31	1.515716567	0.812252396
	IPI00188921	Col1a2	130	1.624504793	0.659753955
	IPI00231662	Cyb5r3	34	1.624504793	0.757858283
	IPI00324380	Ttr	16	1.741101127	0.659753955
	IPI00382098	RT1-EC2	42	2	0.466516496
	IPI00886485	Kng1	49	1.866065983	0.757858283
	IPI00368550	C5	191	0.757858283	1.231144413
	IPI00480639	C3	186	0.659753955	1.624504793
	IPI00202651	Fga	88	1.624504793	0.812252396
	IPI00205389	Fgb	55	0.574349177	1.319507911
	IPI00554059	Serpinc1	67	0.574349177	1.231144413
	IPI00324019	Serpina1	46	1.231144413	0.757858283
	IPI00230783	Nr1d2	43	1.319507911	0.707106781

	IPI00421539	Aco2	85	0.466516496	1.319507911
	IPI00213463	Actn4	105	0.757858283	1.414213562
	IPI00327469	Ahsg	38	1.231144413	0.812252396
	IPI00325146	Anxa2	39	0.757858283	1.515716567
	IPI00207390	Anxa3	36	0.659753955	1.414213562
	IPI00192310	Bcam	68	1.231144413	0.707106781
	IPI00213036	C4b	192	0.707106781	1.624504793
	IPI00191444	Capzb	31	1.515716567	0.812252396
	IPI00470288	Ckb	43	0.707106781	1.624504793
	IPI00189503	Clic5	28	0.707106781	1.319507911
	IPI00205332	Etfa	35	0.812252396	1.231144413
	IPI00470254	Ezr	69	0.812252396	1.515716567
	IPI00205389	Fgb	54	0.812252396	1.414213562
	IPI00231925	Gnai2	41	1.319507911	0.707106781
	IPI00212969	Hnrnpa2b1	34	1.414213562	0.707106781
	IPI00210566	Hsp90aa1	85	0.535886731	1.231144413
	IPI00231136	Nid1	137	0.757858283	1.319507911
	IPI00209000	Plec	534	0.757858283	1.319507911
	IPI00210234	Pls3	71	0.574349177	1.741101127
SAT group	IPI00196684	Prkar2a	46	0.574349177	1.414213562
	IPI00191761	Rab5c	23	0.757858283	1.414213562
	IPI00382098	RT1-EC2	42	2	0.574349177
	IPI00205135	Tgm2	77	1.231144413	0.812252396
	IPI00231139	Tkt	71	0.406126198	1.866065983
	IPI00187731	Tpm2	33	0.812252396	1.624504793
	IPI00231368	Txn1	12	0.812252396	1.319507911
	IPI00212014	Vcp	89	1.319507911	0.812252396
	IPI00230837	Ywhab	28	0.466516496	1.866065983
	IPI00886485	Kng1	49	1.515716567	0.757858283
	IPI00368550	C5	191	0.812252396	1.414213562
	IPI00480639	C3	186	0.757858283	1.515716567
	IPI00554059	Serpinc1	67	0.353553391	1.414213562
	IPI00324019	Serpina1	46	0.812252396	1.231144413
	IPI00230783	Nr1d2	43	1.319507911	0.615572207
	IPI00914765	Zyx	62	2.143546925	0.574349177
	IPI01016329	Ager	14	1.319507911	0.757858283
	IPI00212523	Park7	20	0.659753955	1.319507911
	IPI00327469	Ahsg	39	0.707106781	1.624504793

AT group	IPI00207390	Anxa3	36	0.659753955	1.624504793
	IPI00326305	Atp1a1	113	0.435275282	1.624504793
	IPI00213036	C4b	192	0.707106781	2.143546925
	IPI00768626	Cdh5	87	0.707106781	1.414213562
	IPI00470288	Ckb	43	0.707106781	1.515716567
	IPI00189503	Clic5	28	0.707106781	1.741101127
	IPI00200352	Crip2	23	1.231144413	0.757858283
	IPI00421517	Des	53	1.319507911	0.812252396
	IPI00205332	Etfa	35	0.812252396	1.231144413
	IPI00210542	Gsta4	26	0.5	2
	IPI00557598	LOC100363606	39	1.231144413	0.535886731
	IPI00210234	Pls3	71	0.574349177	1.515716567
	IPI00211779	Prdx1	22	1.319507911	0.435275282
	IPI00324019	Serpina1	46	1.319507911	0.535886731
	IPI00324380	Ttr	16	1.741101127	0.5
	IPI00196684	Prkar2a	46	0.574349177	1.414213562
	IPI00470254	Ezr	69	0.812252396	1.515716567
	IPI00213463	Actn4	105	0.757858283	1.414213562
	IPI00230837	Ywhab	28	0.466516496	1.866065983
	IPI00205135	Tgm2	77	1.231144413	0.812252396
	IPI00209000	Plec	534	0.757858283	1.319507911
	IPI00325146	Anxa2	39	0.757858283	1.515716567
	IPI00324893	Ywhaz	28	1.515716567	0.707106781
	IPI00215465	Cryab	20	0.707106781	1.515716567
	IPI00896184	Slc25a24	53	0.757858283	1.231144413
	IPI00196751	Hspa1b	70	1.231144413	0.659753955
	IPI00231368	Txn1	12	1.231144413	0.757858283
	IPI00212523	Park7	20	1.319507911	0.574349177
	IPI00949898	Hspa8	71	0.659753955	1.414213562
	IPI00190701	Apoe	36	1.414213562	0.812252396
	IPI00190290	Rras2	24	0.757858283	1.319507911
	IPI00198667	Clu	52	0.574349177	1.231144413

**Table 5:** Gene ontology (GO) and pathway enrichment analysis of the DPs.

	Database	Description	Protein number	*P* value
SAT + AT group	GO	Cell-cell adhesion	3	0.036057
	GO	Cadherin binding involved in cell-cell adhesion	3	0.042721
	GO	Cell-cell adherens junction	3	0.047327
	KEGG	Complement and coagulation cascades	8	0.004562
	GO	Complement activation	3	0.014568

SAT group	GO	Actin filament binding	6	2.63*E* − 06
	GO	Actin binding	4	0.007415
	KEGG	Complement and coagulation cascades	6	0.024352
	GO	Complement activation	3	0.014568
	GO	Regulation of inflammatory response	4	0.087112

AT group	GO	Regulation of cell death	4	0.006309
	GO	Negative regulation of hydrogen peroxide-induced cell death	3	0.049772
	GO	Focal adhesion	6	2.39*E* − 04
	GO	Cadherin binding involved in cell-cell adhesion	4	0.005268
	GO	Cell-cell adherens junction	4	0.006171
	GO	Protein complex binding	4	0.021302
	GO	Cell-cell adhesion	3	0.041535
